# Canary Seed (*Phalaris canariensis* L.) Peptides Prevent Obesity and Glucose Intolerance in Mice Fed a Western Diet

**DOI:** 10.3390/ijms232314927

**Published:** 2022-11-29

**Authors:** Uriel Urbizo-Reyes, Andrea M. Liceaga, Lavanya Reddivari, Shiyu Li, Kee-Hong Kim, Abigail D. Cox, Joseph M. Anderson

**Affiliations:** 1 Protein Chemistry and Bioactive Peptides Laboratory, 745 Agriculture Mall Drive, West Lafayette, IN 47907, USA; 2 Department of Food Science, Purdue University, 745 Agriculture Mall Drive, West Lafayette, IN 47907, USA; 3 College of Veterinary Medicine, Purdue University, 625 Harrison Street, West Lafayette, IN 47907, USA; 4 Department of Agronomy, Purdue University, 915 W. State St., West Lafayette, IN 47907, USA

**Keywords:** canary seed peptides, obesity, non-communicable diseases, pancreatic lipase inhibition, hepatic steatosis, glucose tolerance

## Abstract

Previous research showed that canary seed (*Phalaris canariensis* L.) peptides (CSP) possess robust in vitro antiobesity properties via inhibition of pancreatic lipase (PL). Nevertheless, no studies have yet explored their antiobesity properties in vivo. Consequently, we investigated the effects of CSP in C57BL/6J mice under a Western diet (WD). Mice were assigned into groups and fed a normal diet (ND) or a WD accompanied by an oral dose of CSP (250 or 500 mg/kg/day), orlistat (40 mg/kg/day), or distilled water. The results showed that consuming CSP can provide metabolic benefits, including preventing weight gain by up to 20%, increasing glucose tolerance, and reducing insulin, leptin, and LDL/VLDL levels in plasma. Conversely, total ghrelin was unaffected by CSP-500, but decreased by CSP-250, and amplified by orlistat. Surprisingly, CSP-250 was more effective in preventing weight gain and promoting satiety than CSP-500. Parallel to this, protein absorption in CSP-500 was decreased, supported by a rise in fecal crude protein (+3.5%). Similarly, fecal fat was increased by orlistat (38%) and was unaffected by CSP-250 (3.0%) and CSP (3.0%), comparatively to WD (2.5%). Despite this, both CSP treatments were equally effective in decreasing hepatic steatosis and avoiding hyperlipidemia. Furthermore, the enzymatic analysis showed that CSP-PL complexes dissociated faster (15 min) than orlistat-PL complexes (41 min). Lastly, CSP did not affect expression of hepatic lipid oxidation genes ACO and PPAR-α, but reduced the expression of the hydrolase gene LPL, and lipogenesis related genes FAS and ACC. Taken together, these results suggest that CSP antiobesity mechanism relies on lipid metabolism retardation to increase fat transit time and subsequently suppress hunger.

## 1. Introduction

Obesity is a chronic metabolic disorder in which increased adiposity impairs physical and mental health. This condition has reached epidemic proportions globally and has become the major contributor to the global burden of non-communicable diseases (NCDs) [1]. During obesity, adipose tissue stores excess energy in adipocytes cells in the form of triglycerides. As adipose tissue expansion continues, it triggers the production of mediator molecules like adipocytokines or adipokines that give rise to chronic inflammation in various organs and cells [2]. As a risk factor, chronic inflammation is now recognized as an embedded mechanism in the deteriorating function of numerous physiological systems leading to unfavorable alteration in blood pressure, triglycerides, and insulin sensitivity [3]. Remarkably, evidence indicates that the regular consumption of plant-derived bioactive compounds (e.g., phenolic compounds, bioactive peptides (biopeptides), fatty acids, phytochemicals, and sterols) can lower the risk of developing obesity-related complications [4]. In this regard, biopeptides can offer anti-obesity properties via multiple mechanisms like interfering with macronutrient absorption, decreasing adipogenesis, modulating energy expenditure, suppressing appetite, reducing inflammation, and modifying intestinal microbiota, among others [5,6]. Moreover, it’s been established that based on their composition, biopeptides can exert certain bioactivities due to their high specificity, affinity, and efficiency towards specific receptors and enzymes (e.g., α-glucosidase, angiotensin-converting enzyme, α-amylase, and pancreatic lipase) [6]. For instance, soy peptides prevented obesity by activating a leptin-like signaling pathway [7], whereas cocoa peptides exhibit anti-obesity potential by inhibiting pancreatic lipase and preventing NCDs development [8]. Other studies on date seed (*Phoenix dactylifera*) protein hydrolysates showed the antidiabetic capacity of bromelain-generated digests due to their inhibitory activity towards dipeptidyl peptidase-IV (DPP-IV) and α-glucosidase [9].

Recently, hairless canary seed (*Phalaris canariensis* L.), an annual cereal grain, emerged as a novel and safe food ingredient with various nutritional and techno-functional attributes due to its small starch granules, phytochemical profile, and high protein content (19–24%) [10]. Interestingly, over decades, hairless canary seeds have been processed and employed as a traditional medicinal drink, known as “leche de alpiste” (alpiste milk) in Latin American countries to alleviate chronic conditions like cardiovascular diseases, diabetes, hypertension, oxidative damage, and obesity [11,12,13]. In vitro studies have associated the biological activities of canary seed with biopeptides produced during gastrointestinal digestion of the hydrophobic proteins present in the seed [13,14,15]. In this respect, research from our group has unveiled that canary seed peptides produced from commercial proteolysis with Alcalase^®^ have high in vitro bioavailability and are remarkably stable as inhibitors of metabolism-regulating enzymes like pancreatic lipase, angiotensin-converting enzyme (ACE), and dipeptidyl peptidase-IV (DPP-IV) even after simulated gastrointestinal digestion [16]. Although some of the metabolic benefits (e.g., decrease in blood pressure, hypocholesterolemic properties, and hypoglycemic activity) of consuming whole canary seeds have been established in vivo [11,12,17], the studies remain limited, and there is no proposed mechanism of action nor identification of the biologically active compounds associated with the observed health benefits. Whether if canary seed peptides are responsible for the health-promoting responses towards obesity and its pathological disorders remains to be elucidated. Considering the research gaps, the growing incidence of obesity in the westernized population, and the constant pursuit of alternative therapeutic strategies to prevent NCDs, the present study aimed to evaluate the dose–response effects of daily supplementation of isolated canary seed peptides in Western-diet-induced obesity in C57BL/6J mice.

## 2. Results

### 2.1. Effect of CSP on Body Weight, Feed Consumption, and Glucose Tolerance of Mice

**Figure 1:**
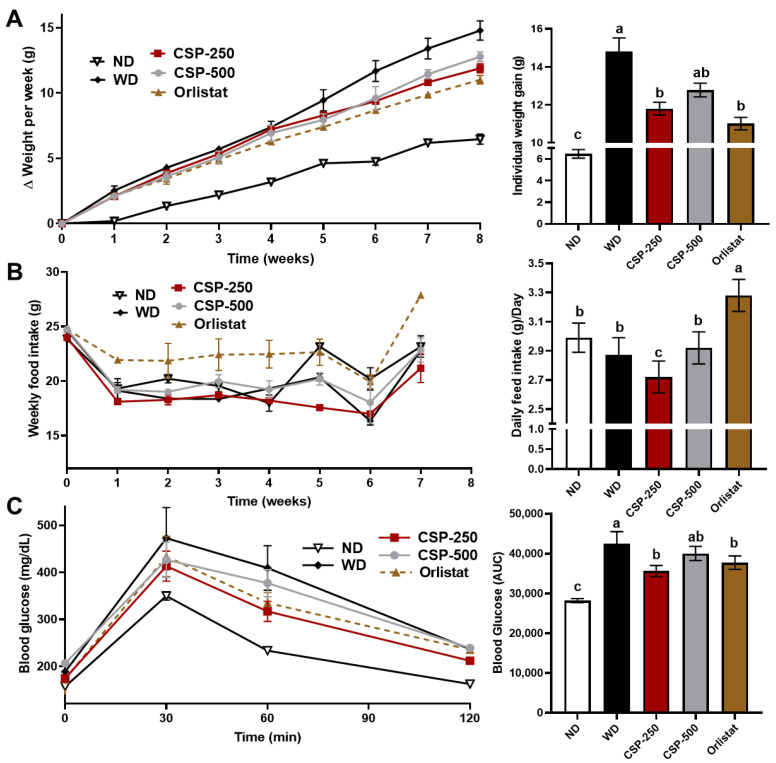
Effect of canary seed peptides (CSP) oral administration over body weight (**A**); weekly and daily feed intake (**B**); and intraperitoneal glucose tolerance in Western diet induced obese C57BL/6J mice at the seventh week of the study and prior to a six-hour fasting period (**C**). ND, normal diet; WD, Western diet; CSP-250, mice supplemented with 250 mg/kg of body weight of CSP; CSP-500, mice supplemented with 500 mg/kg of body weight of CSP; Orlistat, mice supplemented with 40 mg/kg of body weight of orlistat. Bars and lines represent mean values ± SEM (*n* = 8 per treatment). Different letters (a–c) indicate statistical differences (*p* < 0.05) between treatments.

The proximate composition of the CSP powder used in this study comprised of 69.0% crude protein, 18% carbohydrate, 11.4% ash, and 1.3% fat (*w*/*w*, dry basis). In previous studies, we determined that this CSP powder is predominantly rich in low molecular weight peptides with a high degree of hydrolysis (>30%) [15,16]. After eight weeks of study, the daily supplementation of CSP-250 and orlistat, decreased (*p* < 0.05) the body weight gain of mice by 20 and 26%, respectively (Figure 1A). There was no significant (*p* < 0.05) difference in weight gain prevention between CSP-250, CSP-500, and the synthetic drug orlistat. However, at the higher CSP doses (i.e., CSP-500), no difference (*p* > 0.05) in weight gain was found compared to animals fed the WD, indicating a drop in bioactivity at increasing peptide concentrations. As expected, mice fed the ND gained the least body weight after the culmination of the study. Likewise, the feed consumption was also affected, where the highest (*p* < 0.05) daily feed intake (Figure 1B) was observed in the mice supplemented with orlistat (3.3 g/day), and no difference was found between ND (3.0 g/day), CSP-500 (2.9 g/day), and WD (2.9 g/day). Interestingly, the mice supplemented with CSP-250 had the lowest (*p* < 0.05) daily feed intake (2.7 g/day) throughout the study. These results indicate that at low CSP supplementation levels, CSP might also modulate feed intake.

The impact of CSP supplementation on glucose metabolism was assessed by monitoring blood glucose after an intraperitoneal glucose tolerance test (Figure 1C) and determining the mean of the integrated area under the curves (AUC). As expected, mice under WD suffered glucose intolerance, whereas mice under ND displayed the lowest post-glucose challenge AUC values. The AUC for mice under CSP-250 and orlistat was lower (*p* < 0.05) than that observed for mice under WD, but higher than mice fed an ND. The glucose tolerance of animals supplemented with higher peptide concentrations (CSP-500) was similar to CSP-250 and orlistat; however, no difference (*p* > 0.05) was found when compared to mice in the WD group.

### 2.2. Effect of CSP on Lipid Profiles, Organ Weight, and Fecal Proximal Composition

To examine whether the weight gain inhibition of CSP supplementation was associated with adiposity and lipid metabolism, the organ weight and fecal composition were determined at the end of the study (Table 1). As expected, the relative liver and epididymal adipose tissue weight were higher (*p* < 0.05) for mice fed the WD and the lowest (*p* < 0.05) for mice fed an ND. CSP dosages and orlistat successfully lowered relative hepatic weight and epididymal fat deposition to levels similar (*p* > 0.05) of those mice in the ND group. Additionally, the kidney and spleen weights were also evaluated to establish any possible toxic response, and no difference (*p* > 0.05) was found between the CSP, orlistat treatment, and the controls. The fecal composition analysis showed that orlistat supplementation resulted in the highest (*p* < 0.05) fecal fat concentration. In contrast, excreted fecal fat from CSP-supplemented animals was not significantly (*p* > 0.05) different from those of ND and WD groups. Furthermore, crude protein concentration in the fecal matter was significantly (*p* < 0.05) increased by CSP-500 supplementation but not by CSP-250 compared to that of ND and WD animals. Additionally, the fecal concentration of crude protein, crude fiber, and ash was the lowest for the mice supplemented with orlistat (Table 1).

**Figure 2:**
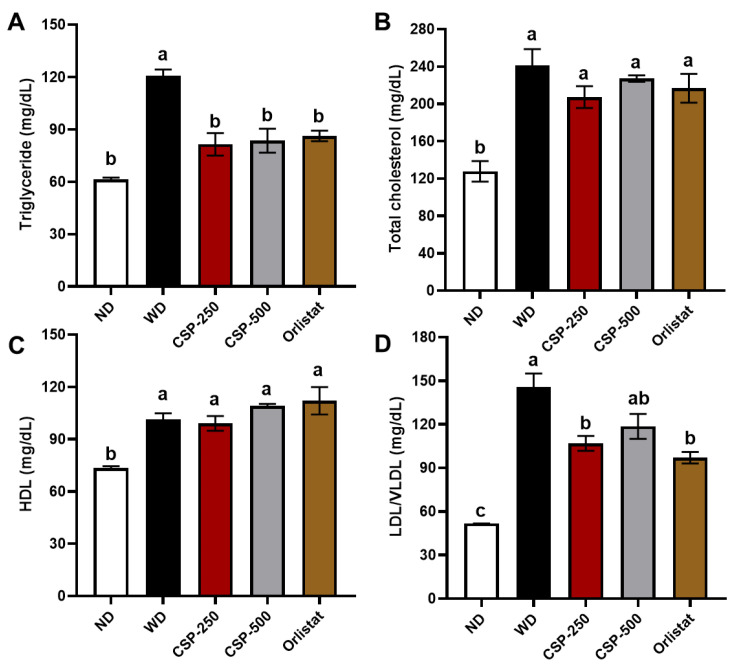
Effect of canary seed peptides (CSP) administration on serum biochemistry after eight weeks of diet-induced intervention in C57BL/6J mice. Fasting serum levels of triglycerides (**A**), total cholesterol (**B**), high density lipoprotein (**C**), and low/very-low density lipoprotein (**D**). ND, normal diet; WD, Western diet; CSP-250, mice supplemented with 250 mg/kg of body weight of CSP; CSP-500, mice supplemented with 500 mg/kg of body weight of CSP; Orlistat, mice supplemented with 40 mg/kg of body weight of orlistat. Bars and lines represent mean values ± SEM (*n* = 8 per treatment). Different letters (a–c) indicate statistical differences (*p* < 0.05) between treatments.

The serum TG, HDL, LDL/VLDL, and total cholesterol levels were the highest (*p* < 0.05) in the WD group and lowest for the ND group (Figure 2). In addition, CSP-250, CSP-500, and orlistat supplementation were effective in lowering (*p* < 0.05) TG concentration in serum to similar levels of mice in the ND group (Figure 2A). Similarly, CSP-250 and orlistat groups displayed a reduction (*p* < 0.05) in the serum LDL/VLDL concentration compared to WD but were still higher than the ND group. Conversely, no significant difference (*p* > 0.05) was found in LDL/VLDL serum concentrations between CSP-500 and WD groups. Likewise, no difference (*p* > 0.05) in HDL and total cholesterol was found between CSP-250, CSP-500, orlistat, and the WD group. These results indicate that daily CSP supplementation can be an effective alternative for preventing hyperlipidemia and improving blood cholesterol profiles.

### 2.3. Modulatory Effect of CSP on Metabolic Hormones Levels and Pancreatic Lipase Activity

**Figure 3:**
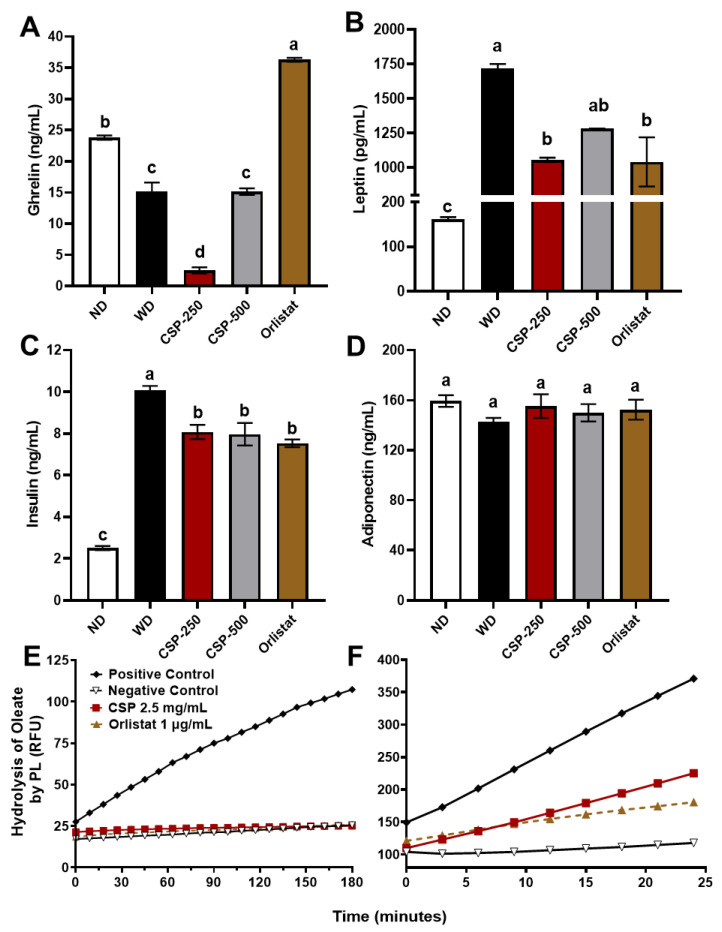
Effects of canary seed peptides (CSP) administration on regulation of metabolic hormones after eight weeks of diet-induced intervention in C57BL/6J mice and inhibition against pancreatic lipase (PL). Fasting serum levels of ghrelin (**A**), leptin (**B**), insulin (**C**), and adiponectin (**D**) hormones. Complete inhibition of PL by CSP and orlistat (**E**). 20-fold dilution of PL-inhibitor complex with oleate promotes recovery of PL activity from PL-CSP complexes (**F**). ND, normal diet; WD, Western diet; CSP-250, mice supplemented with 250 mg/kg of body weight of CSP; CSP-500, mice supplemented with 500 mg/kg of body weight of CSP; Orlistat, mice supplemented with 40 mg/kg of body weight of orlistat. Bars and lines represent mean values ± SEM (*n* = 8 per treatment). Different letters (a–d) indicate statistical differences (*p* < 0.05) between treatments.

The effect of CSP on metabolic hormones and pancreatic lipase activity is shown in Figure 3. At the end of the study, serum leptin and insulin levels were the highest (*p* < 0.05) for the WD group and the lowest for mice under ND. Meanwhile, animal groups supplemented with CSP-250 and orlistat exhibit a reduction (*p* < 0.05) in leptin and insulin levels. Additionally, when compared to the WD group, CSP-500 also decreased insulin levels significantly (*p* < 0.05) but not leptin concentrations (*p* > 0.05). Interestingly, CSP-250 supplementation, but not CSP-500, induced a significant (*p* < 0.05) reduction of serum ghrelin concentrations compared to the controls. Conversely, orlistat supplementation increased (*p* < 0.05) the concentration of serum ghrelin. These results aligned with the feed intake observations reported earlier, were animals in the CSP-250 diet group displayed the lowest daily feed intake and animals from the orlistat group had the highest daily feed intake (Figure 1B).

The regulation of pancreatic lipase by CSP and orlistat is shown in Figure 3E,F, respectively. The pancreatic lipase-inhibitor complexes were pre-formed to inhibit the enzyme activity by completely saturating the enzyme with 10 × IC_50_ based on our previous studies [15,16]. The complexes were stable and inactive (Figure 3E), presenting no product formation, and were monitored for three hours. The residence time of the inhibitors was determined after performing a 30-fold jump dilution experiment with 4-methylumbelliferyl oleate. The results showed rapid recovery of pancreatic lipase activity by CSP inhibitor dissociation and a slow recovery in pancreatic lipase activity from orlistat complexes (Figure 3F). The residence times for CSP and orlistat were 15 and 41 min, respectively. These results demonstrate the reversible inhibitory nature of CSP towards pancreatic lipase and the slow reversibility of orlistat once bound to pancreatic lipase.

### 2.4. Effect of CSP on Liver Histopathology, Lipid Metabolism, and Gene Expression

**Figure 4:**
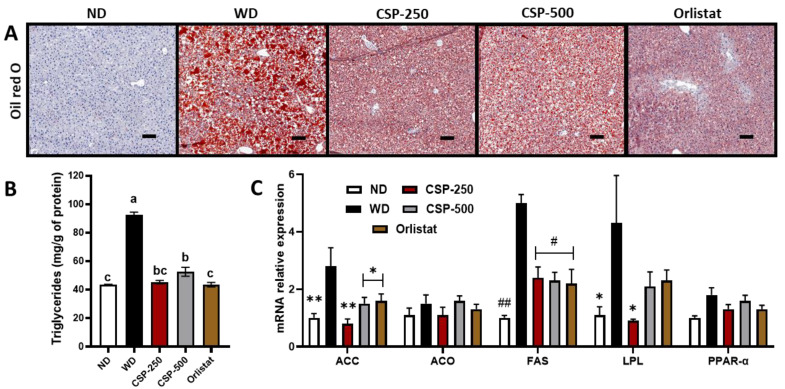
(**A**) Prevention of liver steatosis by daily administration of canary seed peptides (CSP) for an eight-week diet induce intervention in C57BL/6J mice. Magnified histological liver sections (200×) stained with hematoxylin-eosin Oil red O (Scale bar, 100 μm for all). (**B**) Triglycerides levels and, (**C**) lipid metabolism related gene expression on hepatic tissue. ND, normal diet; WD, Western diet; CSP-250, mice supplemented with 250 mg/kg of body weight of CSP; CSP-500, mice supplemented with 500 mg/kg of body weight of CSP; Orlistat, mice supplemented with 40 mg/kg of body weight of orlistat. Bars and lines represent mean values ± SEM (*n* = 8 per treatment). Different letters (a–c) indicate statistical differences (*p* < 0.05) between treatments. * *p* < 0.05, ** *p* < 0.05, # *p* < 0.005, and ## *p* < 0.0001 vs. WD group.

To further explain the changes in liver weight between treatments (CSP and orlistat) and WD reported in Table 1, and to further explore the protective effect of CSP in liver pathophysiology, histology, TG, and gene expression analysis was carried out (Figure 4). TG analysis revealed that both CSP dosages and orlistat were successful in reducing (*p* < 0.05) the hepatic TG deposition (steatosis) and had similar levels to those of mice in the ND group. These observations were further confirmed by microscopic examination of the liver tissue under Oil red O staining for neutral lipids (Figure 4A). Two conditions became apparent: macrovesicular steatosis (large TG droplet vacuole filling the hepatocyte) in the WD group and microvesicular steatosis (small intracytoplasmic TG vacuole filling the hepatocyte) in CSP and orlistat treatments (Supplementary Materials Table S1). No difference (*p* > 0.01) was observed in the inflammatory parameters such as ballooning and lobular inflammation when evaluated microscopically under H&E staining.

In addition, hepatic gene expression analysis (Figure 4C) showed a parallel trend in lipid metabolism genes between mice in the CSP and orlistat diet groups. Particularly, CSP and orlistat supplementation resulted in significant downregulation of fatty acid synthesis genes such as FAS and ACC. Meanwhile, genes involved in lipid oxidation (ACO and PPAR-α) remained unchanged compared to the WD group. Moreover, only the ND and CSP-250 diet groups exhibited a significant suppression in the expression of the LPL encoding gene.

## 3. Discussion

In our previous in vitro studies, we established that CSP had a high potency in inhibiting pancreatic lipase and that after gastrointestinal digestion, its potency increased 10-fold [15,16]. In addition, we previously showed that CSP inhibited pancreatic lipase uncompetitively by destabilizing the open-lid conformation via arginine and non-polar residues interactions [16]. It is worth mentioning that CSP are less potent and stable than synthetic pancreatic lipase inhibitors like orlistat. Therefore, higher concentrations of CSP were tested to elucidate any possible biological effects in this study. Henceforth, the present study explored the potential therapeutical benefits of CSP consumption in preventing obesity and its metabolic consequences. Our results demonstrated that C57BL/6J mice fed a WD and supplemented daily with CSP exhibited lower weight gain, decreased feed intake, and increased glucose tolerance compared to mice fed the WD alone. Furthermore, the CSP metabolic benefits observed in this study were similar to those of the commercial antiobesity drug orlistat. In this respect, it is recognized that orlistat effectively inhibits gastric and pancreatic lipases, preventing the breakdown of dietary fats into absorbable triglycerides, free fatty acids, and monoglycerides, hence stopping fat absorption and increasing the fecal fat concentration [18,19,20]. Yet, it’s been reported that undesired side effects might accompany treatment with synthetic drugs like orlistat (e.g., oily spotting, abdominal cramping, fecal incontinence, and fat-soluble vitamin deficiencies) [21]. In this study, CSP’s potency was inferior to that of the commercial drug orlistat; however, the side effects like steatorrhea were not triggered by the peptides. Hence, studying natural inhibitors (e.g., polyphenols, peptides, flavonoids, saponins, terpenoids, alkaloids) from plants with fewer side effects is of high interest in nutraceutical research [22].

Surprisingly, our study showed that CSP-250 was more effective at preventing weight gain than CSP-500, with the latter being not different from the WD group. Likewise, CSP-250 but not CSP-500 decreased feed intake and ghrelin expression, yet both CSP treatments exhibited high hypolipidemic and anti-steatosis effects. Moreover, the drop in weight gain prevention capacity by CSP-500 could be attributed to the saturation of tolerable dietary protein intake per meal in the small intestine, leading to indigestion of the peptides. While no studies have yet suggested a maximum absorbable protein load (protein content/kg of BWT/meal) in mice, recent research in humans suggests that optimal protein consumption should range between 0.33–0.40 g of protein/kg of BWT/meal and that higher protein levels may result in a rise in amino acid oxidation, and indigestion of proteins causing an increase in fecal protein concentration [23,24,25]. Other studies in pigs have reported similar observations, where a significant reduction of protein digestibility and an increase of fecal crude protein resulted from increased dietary protein levels’ supplementation from 12 to 18% in ad libitum diets [26]. In agreement with this, the protein content in fecal matter from CSP-500 treated animals was higher than that of the controls and CSP-250 animals. However, additional studies are needed to fully understand the maximum tolerable protein load per meal/dosage in mice and the effect of CSP-500 when distributed into several doses throughout the day.

Considering that CSP-250 displayed hypolipidemic activity, satiety properties, and an absence of steatorrhea, this suggests that CSP-250 functioned through a dual mechanism to prevent obesity. It is hypothesized that a delay in lipid metabolism might account for the satiety and hypolipidemic observations in the CSP-250 animals. With this in mind, the enzymatic study revealed that the residence time of CSP over pancreatic lipase enzyme was 2.7-fold shorter than that of orlistat. Hence, shorter inhibitor residence times are known to translate into a faster dissociation of enzyme-inhibitor complexes in vivo [27,28] and, in this case, delay but not completely inhibit the digestion of fats. In this respect, studies have reported that the presence of fatty acids and monoacylglycerol in the duodenal section of the intestine has more satiating effects than unhydrolyzed fats. Thus, compounds that reduce dietary fat absorption rates are valuable strategies for inducing satiety [29,30]. For instance, Dimethylaminoethyl dodecyl ether, a non-hydrolysable lipid compound that delays but does not inhibit fat digestion by binding to pancreatic lipase, had a more satiating effect than compounds yielding unhydrolyzable fat in the intestine such as orlistat [31]. Similarly, researchers found that dietary supplementation of cell membranes from plants, animals, and bacteria decreased the rate of lipase/colipase catalyzed hydrolysis of TG, and when supplemented in combination with refined foods, the compounds suppressed food intake in rats by retardation of fat digestion [32]. Consistent with the results of this study, Ojeda et al. [33] evaluated the metabolic effect of a 25% canary seed infusion on rats under a hypercaloric diet for 45 days, where rats that received a whole canary seed infusion combined with a dietary change showed a reduction of 30% in TG values. Similarly, Almaraz et al. [34] found that both aqueous and hexane canary seed extracts successfully prevented metabolic syndrome indicators like hypertriglyceridemia. In agreement with this, we previously reported that canary seeds are predominantly rich in hydrophobic protein fractions called prolamins [15]. Subsequently, we fractionated the canary seed proteins and identified four peptides (e.g., VPPR, LADR, LSPR, and TVGPR) with high inhibitory activity towards pancreatic lipase. Taken together, it is possible that the dissociation of CSP-pancreatic lipase complexes played a critical role in reducing serum TG, adipose tissue deposition, and in the case of CSP-250, preventing weight gain by inducing satiety. While the health benefits of canary seeds have been previously reported, this is the first time that a proposed mechanism has been established using isolated canary seed peptides as bioactive compounds.

Obesity is also associated with an increased risk of non-alcoholic fatty liver disease (NAFLD), characterized by hepatic steatosis (intrahepatic fat of at least 5% of liver weight). Hepatic steatosis occurs when the uptake of fatty acid from plasma and the de novo fatty acid synthesis exceeds the rate of fatty acid oxidation and metabolism, and its progression can lead to chronic liver disease, Type-2 diabetes, and cardiovascular disease [35,36]. Traditionally, treatments of NAFLD included pharmacological interventions (e.g., metformin, incretins, thiazolidinediones, antiobesity drugs) and lifestyle modification (e.g., weight loss programs, change in diet, exercise) [37]. In addition, studies have also validated the efficacy of orlistat administration in treating NAFLD. For instance, a 6-month clinical trial involving 191 patients showed that 53% of the patients treated with orlistat improved their liver steatosis condition compared to routine treatments involving dietary and physical activity interventions [20]. In agreement with this, orlistat as well as CSP treatments were successful in the reduction of hepatic steatosis as well as reducing LDL/VLDL levels. Thus, we studied the CSP-mediated effect on lipid synthesis, storage, and metabolism genes. It was found that CSP suppressed the gene expression of FAS and ACC, transcription factors important in lipogenesis, as well as the LPL encoding gene, a transcription factor involved in lipid metabolism [36]. The role of FAS has been established as a critical multifunctional enzyme responsible for catalyzing the palmitate synthesis pathway and increasing De novo lipogenesis in the body, liver, and adipose tissue [38]. Likewise, ACC also promotes lipogenesis by mediating the catalysis of acetyl-CoA into malonyl-CoA, a two-carbon donor unit essential for the synthesis of palmitate fatty acids [39]. Recent studies have also established that increased upregulation of hepatic LPL is strongly associated with NAFLD due to its rate-controlling function in plasma triglyceride hydrolysis and incorporation into tissues [40,41]. In this study, CSP had no modulatory activity over transcription factors PPAR-α and ACO, genes involved in the metabolism and oxidation of fatty acids [42]. It is known that PPAR-α act as one of the master regulators responsible for translating nutritional and metabolic stimuli for fat oxidation genes such as Pdk4, Fgf21, ACO, and Cpt1α [38,43]. At the same time, ACO functions as a rate-limiting enzyme involved in the first step of fatty acid peroxisomal β-oxidation [42]. Overall, this study suggests that the anti-hepatic steatosis effect was likely attributed to a reduced systemic lipid uptake and deposition rather than enhanced lipid oxidation. Additionally, it was established that CSP has exceptional anti-hyperlipidemic properties and that at moderate concentration, it might prevent weight gain via retardation of lipid metabolism and hunger suppression. In agreement with these observations, Perez Gutierrez et al. [44] showed how hexane extracts from canary seeds decreased weight gain in the liver and reduced triglyceride deposition in hepatic tissue. Furthermore, they also found that the extracts effectively decreased transcriptional factors associated with oxidative stress like SOD, CAT, and CSH. Nevertheless, future studies should now be geared towards determining CSP’s effect on oral lipid tolerance and its long-term impact on fat-soluble vitamin absorption compared to synthetic drugs.

## 4. Materials and Methods

### 4.1. Preparation of Canary Seed Peptides (CSP)

USDA-GRAS hairless canary seeds (CDC Cibo) were purchased from a commercial vendor (Canpulse Foods LTD, Saskatoon, SK, Canada). As previously indicated, canary seeds were subject to mechanical oil removal [15]. Then, the defatted canary seed flour was subject to isoelectric protein extraction following the methodology proposed by Achouri et al. [45] with slight modifications. Briefly, defatted canary seed flour was suspended in distilled water (1:15 ratio, protein: liquid), and the pH was adjusted to 10 using 4 M NaOH, followed by constantly stirring for one hour at 22 ± 3 °C. The protein-rich fraction (supernatant) was recovered by centrifugation at 1000× *g* for 20 min, and then the pH was adjusted to 4 using 1 N HCl. A second centrifugation step was used to precipitate the canary seed protein. The isolated protein was then suspended in distillated water (22.5 mg of protein/mL at pH 8) and subject to proteolysis [16]. The reaction was carried out at 50 ± 3 °C for four hours with 3% (*w*/*w*) food-grade Alcalase^®^ (Protease of *Bacillus licheniformis*, P2.4 U/g; Novozymes, Bagsvaerd, Denmark). Proteolysis was terminated by pasteurization (95 ± 3 °C) for 15 min. Canary seed peptides were recovered from the supernatant by centrifugation (17,636 g for 15 min) (Avanti J-26S Centrifuge, Beckman-Coulter Inc., Brea, CA, USA), frozen (−80 °C), and lyophilized using a Labconco FreeZone Plus 2.5 L cascade benchtop freeze dry system (Labconco Corp., Kansas City, MO, USA). Canary seed peptide powder was composed of 70% crude protein, 1.3% crude fat, 17% carbohydrate, and 11.4% ash dry basis. Finally, CSP’s proximate composition was determined, as we previously described [46], through a commercial analytical laboratory (A&L Great Lakes, Fort Wayne, IN, USA).

### 4.2. Animals and Housing

Four-week-old male C57BL/6J mice (*n* = 40) were purchased from the Jackson Laboratory (Bar Harbor, ME, USA). All the experimental animal procedures were approved by Purdue University Animal Care and Use Committee [PACAUC approval No.1810001817A003] according to the recommendations of the Guide for the Care and Use of Laboratory Animals published by the National Institutes of health. The mice had ad libitum access to water and food and were housed under 12 h light/dark cycles at 23 ± 3 °C and controlled humidity 50–60% throughout the experimental period.

### 4.3. Experimental Design, Diets, and Sample Collection

Following arrival, mice were acclimatized for ten days and then randomly allocated into the following five groups (*n* = 8 per group): normal diet (ND) group, mice were fed a standard diet + saline solution; Western diet (WD) group, mice were fed a western-style diet + saline solution; canary seed peptide-250 (CSP-250) and 500 (CSP-500) groups, mice were fed a western-style diet + 250 or 500 mg CSP/kg of body mass solution; and orlistat group, mice were fed a western-style diet + 40 mg orlistat/kg of body mass solution. Distilled water, CSP, and orlistat solutions were supplemented daily in equivalent volumes by oral gavage for the eight weeks of the study. The standard diet (TD.05230, with 4% of fat calories) and western-style diet (TD.88137, with 42% of fat calories) were purchased from Envigo (Madison, WI, USA), and their formulation and caloric composition are given in Table 2.

The total body weight gain (g/animal), daily feed intake (g/animal/day), and water consumption was monitored weekly throughout the study. The fecal collection was done per cage, separating fecal matter from bedding on a weekly basis and then stored at −80 ± 3 °C until analysis. After the eight weeks of the study, animals were sacrificed by CO_2_ asphyxiation. Immediately after, blood was collected by cardiac puncture. Finally, kidney, spleen, liver, and epididymal fat were weighted and stored at −80 ± 3 °C.

### 4.4. Intraperitoneal Glucose Tolerance Test (IPGTT)

The glucose tolerance test was conducted at the seventh week of the study, following the previously described methodology by Small et al. [47]. Briefly, each mouse underwent a fasting period of six hours. Then, blood samples were collected to quantify circulating glucose by making a small incision at the tip of the tail and analyzed using a NovaMax glucometer (Nova Diabetes Care, Billerica, MA, USA). Blood glucose was quantified at baseline and after (30, 60, and 120 min) an intraperitoneal injection of a 45% D-(+)-Glucose solution (2 g of glucose/kg body mass). Finally, the results were analyzed and reported as an area under the curve (AUC).

### 4.5. Fecal Proximal Composition

Moisture, crude protein, crude fat, crude fiber, of the fecal matter were determine by the official methods of analysis AOAC 934.01, AOAC 990.03, AOCS Am5-04, AOCS Ba6a-05 by a commercial laboratory, Waters Agricultural Laboratories (Camilla, GA, USA).

### 4.6. Serum Biochemical Analysis

Total serum high-density lipoprotein (HDL), low-density lipoprotein (LDL)/very low-density lipoprotein (VLDL), and total cholesterol levels were quantified with HDL, and LDL/VLDL Colorimetric/Fluorometric assay Kit (LSK314100) obtained from Lifespan Bioscience (Seattle, DC, USA). The assays were carried out following the manufacturer’s instructions, and the absorbance was measured using Multiskan™ FC Microplate Photometer (Waltham, MA, USA). In addition, serum triglyceride (TG) levels were measured using a triglyceride colorimetric kit (LSK302200) purchased from Lifespan Bioscience (Seattle, DC, USA).

### 4.7. Measurement of Fasting Serum Levels of Metabolic Hormones

Mouse serum was analyzed to quantify total serum ghrelin using a mouse enzyme-linked immunosorbent assay (ELISA) ghrelin kit (EIAM-GHR-1) obtained from Raybiotech (Norcross, GA, USA), serum leptin by mouse leptin Duoset ELISA kit (DY49805) obtained from R&D Systems (Minneapolis, MN, USA), serum adiponectin and insulin by commercial mouse ELISA kits (KMP0041 and EMINS) obtained from Thermo Fisher Scientific (Waltham, MA, USA). All assays were carried out following the manufacturer’s instructions. Finally, the absorbances were quantified at 450 nm and turbidity corrections at 590 nm using Multiskan™ FC Microplate Photometer (Waltham, MA, USA).

### 4.8. Pancreatic Lipase Inhibition

Pancreatic lipase inhibition was measured according to the procedures described in our previous study [16]. Briefly, samples, substrate, and pancreatic lipase were dissolved in McIlvaine buffer (0.1 M Citrate-Na2HPO4, pH 7.4). Then, 50 µL of substrate 4-methylumbelliferyl oleate (1 mM) was pre-incubated with 25 µL of peptide sample for 10 min in a black 96-well microplate. Then, 25 µL of pancreatic lipase type-VI (200 U/mL) were pipetted, and the reaction was carried out for one hour. The production of 4-methylumbelliferone was measured fluorometrically using a Fluoroskan Ascent FL Microplate Fluorometer and Luminometer, ThermoFisher Scientific (Waltham, MA, USA) using an excitation wavelength of 355 nm and emission wavelength of 460 nm. The positive control (no inhibitor) used the buffer instead of the sample, and buffer was used instead of pancreatic lipase solution for negative control (no pancreatic lipase activity). Finally, a jump dilution experiment was carried out, as previously reported by Kumar and Lowery [48]. Briefly, enzyme-inhibitor complexes were formed by pre-incubating the substrate, pancreatic lipase (6000 U/mL), and inhibitors (10 × IC50) for three hours. Then, the activity recovery was measured after performing a 30-fold jump dilution, and the reaction was monitored for 25 min. Finally, the residence time was calculated by measuring tau determined as the reciprocal of koff after fitting the enzyme progress curves to Equation (1).

(1) $$Abs=V_{s}t+\left(V_{0}-V_{s}\right)\frac{\left(1-e^{-k_{off}t}\right)}{k_{off}}$$

### 4.9. Histopathology

Liver sections were embedded in paraffin blocks, sliced [4 µm], and stained using standard hematoxylin and eosin staining, followed by microscopically evaluation. Similarly, oil red O staining was carried out by embedding in optimal cutting temperature gel, and the samples were cut to a thickness of 6 µm sections. The sections were quickly submerged in formalin and stained with 0.5% oil red O solution. All sides were prepared and evaluated by a board-certified veterinary pathologist from the Histology Research Laboratory at Purdue University. The hepatic histological scores were achieved by following the histomorphological scale (Supplementary Materials Table S2).

### 4.10. Hepatic Triglyceride Analysis

Triglycerides from mouse liver were extracted and quantified as proposed by Adhyatmika et al. [49], and Smith et al. [50]. Briefly, hepatic tissue was homogenized for 40 s in 1 mL of extraction buffer that contained 25 mM Tris, 10 mM sodium phosphate, 150 mM NaCl, 0.1% SDS, 1% Triton-X100, and protease inhibitor. The tissue sample was then diluted with 5 mL of a chloroform/methanol (2:1, *v*/*v*) solution vortex briefly and incubated under an ice bath for two hours. The solution was centrifuged at 1650× *g* for 10 min at 4 °C, and three phases were identified. The upper phase was collected and subject to a second extraction step by adding 600 µL of 4 mM MgCl_2_ and vortexed vigorously and incubated under an ice bath for 30 min, followed by centrifugation 1650× *g* for 10 min at 4 °C. The bottom phase from the first extraction was combined with the bottom phase of the second extraction. The chloroform was removed by evaporation under nitrogen gas in a 37 °C water bath. The triglycerides were suspended with 200 µL of distillate water and vortexed vigorously to create the final emulsion. Finally, the triglyceride content was quantified using the Infinity Triglyceride Reagent (Thermo Scientific, Waltham, MA, USA) following manufacturer’s instructions and normalized to protein content.

### 4.11. Gene Expression by Quantitative Real-Time Polymerase Chain Reaction (qPCR)

RNA was extracted following the methodology proposed by Siersbæk et al. [51], and qRT-PCR analysis following the proposed method by Pfohl et al. [52]. Briefly, 15 mg of liver tissue were homogenized using TRIzol-RNA lysis reagent (Invitrogen) for 5 min. The RNA was then purified by adding 150 μL of chloroform followed by centrifugation at 12,000× *g* for 15 min at 4 °C. The supernatant was recovered as a source of RNA and was further washed and centrifuged three times with 0.5 mL of 75% ethanol at 12,000× *g* for 3 min. Lastly, the pellet was resuspended with 0.5 mL of DPEC water, 0.5 mL of isopropanol, and 50 µL of a 3 M sodium acetate solution at pH 5.5 followed by centrifugation at 12,000× *g* for 15 min at 4 °C. The precipitated RNA was then suspended in Diethyl pyrocarbonate (DEPC) water, and complementary DNA (cDNA) synthesis using the superscript II kit according to the manufacturer’s protocol. The cDNA was amplificated using SYBR premix Plus (SYBR Green) (Bio-Rad Laboratories, Hercules, CA, USA). The reaction was carried out using StepOne Real-Time PCR System (Applied Biosystems, Foster City, CA, USA), and the expression was normalized to the housekeeping gene GAPDH. Lastly, the data were analyzed using the Delta Delta CT (2^–∆∆Ct^) method to calculate the fold gene expression change relative to the control (ND). Primers used to quantify each transcript are specified in Supplementary Materials Table S3.

### 4.12. Statistical Analysis

The results in this study were analyzed using a randomized complete block design by a one-way-ANOVA followed by a Tukey’s and Fisher’s LSD multiple comparison test (*p* < 0.05), where blocks were animal cages. Data analysis was done using the statistical software statistical software SAS^®^ version 9.4 (SAS Institute, Cary, NC, USA) and GraphPad Prism^®^ version 9.4.0 (GraphPad, San Diego, CA, USA). Finally, the results were reported as mean ± standard error of the mean (SEM).

## 5. Conclusions

In conclusion, administration of canary seed peptides (CSP) generated by commercial proteolysis suppressed metabolic consequences derived from obesity such as weight gain, hyperlipidemia, glucose tolerance, and hepatic steatosis of C57BL/6J mice fed a Western diet. It is worth mentioning that moderate supplementation of CSP (i.e., 250 mg/kg of body weight) was more effective in preventing weight gain than the high CSP dose (i.e., 500 mg/kg of body weight), preventing weight gain. Moreover, CSP supplementation reduced steatorrhea compared to the commercial drug orlistat. Likewise, CSP promoted a drop in fatty acid uptake gene, LPL, and fatty acid biosynthesis genes FAS and ACC while unaffecting lipid oxidation genes PPAR-α and ACO. The regulation of these genes is related to a lower fat-derived fuel utilization and decreased bloodstream lipid uptake. Moreover, this study suggests that CSP’s weight gain inhibition depended on a dual mechanism involving a delay in lipid metabolism and satiety to modulation. Supporting this theory, in vitro enzymatic analysis showed the rapid dissociation capacity of CSP-pancreatic lipase complexes and the critical role of its binding reversibility in reducing serum TG absorption rates and inducing satiety. Lastly, limited information is available concerning the effect of isolated canary seed protein or CSP on obesity-related complications and their possible mechanism of action. Despite this, our findings describe CSP’s possible mechanism of action and support their potential use as an effective nutraceutical for functional foods and as an antiobesity treatment.

## Figures and Tables

**Table 1:** Proximal composition of fecal matter, organ and epididymal fat weight as percentage of body weight in C57BL/6J mice after 8 weeks of intervention.

Treatment Group	Fecal Proximal Composition (% in Dry Weight)					Organ and Tissue Weights			
	Crude Protein	Crude Fat	Crude Fiber	Nitrogen Free Extract	Ash	Kidney (mg)	Spleen (mg)	Liver (%, w/bwt)	Epididymal Fat (%, w/bwt)
Normal diet	17.64 ± 0.04 ab	2.23 ± 0.30 b	33.08 ± 0.93 a	47.48 ± 0.93 b	20.27 ± 0.63 a	371.64 ± 4.36 a	86.27 ± 3.50 a	4.03 ± 0.06 b	3.07 ± 0.27 d
Western diet	15.29 ± 0.05 c	2.58 ± 0.01 b	28.9 ± 1.21 a	47.56 ± 1.21 b	21.85 ± 0.16 a	390.76 ± 0.76 a	85.25 ± 0.50 a	5.42 ± 0.48 a	6.68 ± 0.20 a
CSP-250	16.14 ± 0.01 bc	3.03 ± 0.01 b	30.41 ± 1.06 a	48.02 ± 1.06 b	21.04 ± 0.41 a	386.50 ± 10.25 a	80.4 ± 0.93 a	4.70 ± 0.01 ab	5.97 ± 0.12 bc
CSP-500	18.83 ± 0.91 a	3.02 ± 0.39 b	31.58 ± 0.80 a	48.74 ± 0.80 b	20.34 ± 0.63 a	369.08 ± 12.83 a	84.60 ± 3.70 a	4.61 ± 0.25 ab	6.28 ± 0.45 ab
Orlistat	12.62 ± 0.15 d	38.67 ± 0.16 a	19.9 ± 0.32 a	93.42 ± 0.32 a	13.1 ± 0.19 b	402.08 ± 7.43 a	81.50 ± 0.50 a	4.46 ± 0.29 ab	5.41 ± 0.32 c

Values represent mean values ± SEM (*n* = 8 per treatment). Different letters (a–d) within a column indicate statistical differences (*p* < 0.05) between treatments. Bwt: body weight.

**Table 2:** Proximal composition (wet basis) and formulation of experimental diets.

Macro-Nutrient	Normal Diet	Western Diet
	TD.05230	TD.88137
Protein (%)	17.3	17.3
Carbohydrate (%)	63.5	48.5
Fat (%)	5.2	21.2
Energy (kcal/g)	3.7	4.5
**Ingredient**	**Formula (g/kg)**	
Casein	195	195
DL-methionine	3	3
Sucrose	341	341
Corn Starch	212	150
Maltodextrin	100	N/A
Anhydrous Milkfat	37	210
Soybean oil	13	N/A
cholesterol	N/A	1.5
cellulose	50	50
Mineral mix, (AIN-76)	35	35
Calcium Carbonate	4	4
Vitamin Mix (Teklab-40060)	10	10
Ethoxyquien-antioxidant	0.01	0.04

## Data Availability

Data is contained within the article or supplementary material.

## Supplementary Materials

The following are available online at https://www.mdpi.com/article/10.3390/ijms232314927/s1, Reference [53] are cited in the supplementary materials.
