# Supplementary material for: Canary Seed (Phalaris canariensis L.) Peptides Prevent Obesity and Glucose Intolerance in Mice Fed a Western Diet
Source: Int J Mol Sci. 2022 Nov 29;23(23):14927. doi: 10.3390/ijms232314927 (PMC9736008; doi:10.3390/ijms232314927)
Supplement: Supplementary file 1 [file ijms-23-14927-s001.zip › ijms-2041416-supplementary.pdf]

Supplementary Materials

Canary Seed (*Phalaris canariensis* L.) Peptides Prevent Obesity and Glucose Intolerance in Mice Fed a Western Diet

Uriel Urbizo-Reyes [1,2], Andrea M. Liceaga [1,2,*], Lavanya Reddivari [2], Shiyu Li [2], Kee-Hong Kim [2], Abigail D. Cox [3] and Joseph M. Anderson [4]

[1] Protein Chemistry and Bioactive Peptides Laboratory, 745 Agriculture Mall Drive, West Lafayette, IN 47907, USA
[2] Department of Food Science, Purdue University, 745 Agriculture Mall Drive, West Lafayette, IN 47907, USA
[3] College of Veterinary Medicine, Purdue University, 625 Harrison Street, West Lafayette, IN 47907, USA
[4] Department of Agronomy, Purdue University, 915 W. State St., West Lafayette, IN 47907, USA
* Correspondence: aliceaga@purdue.edu

Table S1. Qualitative assessment of the vesicle size of each ORO slide.

Mouse	Macro-vesicular	Micro-vesicular	Negligible	PHOTO
Orlistat_3 c2	x	x		
Western_1 c2	x			
250_2 c1		x		x
250_3 c1		x		
250_0 c1		x		
250_1 c1	x	x		
250_2 c2	x	x		
orlistat_0 c2		x		
500_2 c2		x		
250_1		x		
lowcal_1 c1			x	
orlistat_1 c1			x	
western_3 c1	x	x		
western_0 c2	x			
Western_1 c1	x			
western_0 c1	x			x
lowcal_3 c1			x	
lowcal_1 c2			x	
500_3 c2	x			
orlistat_2 c2			x	

lowcal_0 c2			x	
lowcal_2 c1			x	x
500_2 c1		x		
500_3 c1		x		
lowcal_2 c2		x		
orlistat_3 c1			x	
orlistat_1 c2		x		
lowcal_0 c1			x	
250_3 c2	x	x		
orlistat_2 c1		x		x
western_2 c2	x			
500_0 c1			x	
lowcal_3 c2		x		
western_3 c2	x			
orlistat_0 c1			x	
500_1 c1		x		x
250_0 c2	x			
western_2 c1	x			
500_0 c2		x		
500_1 c2	x			

Table S2. Histological criteria of examination.

Item	Definition	Score
Steatosis	Macro-vesicular	Qualitative
	Micro-vesicular	Qualitative
	negligible	Qualitative
Liver cell injury	None	0
	Few balloon cells (rare but definite)	1
	Many cells/prominent ballooning	2
Lobular inflammation (200x)	No foci	0
	<2 foci per 200X field	1
	2-4 foci per 200x field	2
	>4 foci per 200x field	3

Adapted fronm Kleiner, David E., et al. "Design and validation of a histological scoring system for nonalcoholic fatty liver disease." Hepatology 41.6 (2005): 1313-1321.

Table S3. Primer sequences used for hepatic gene expression analysis.

Primer	Forward Sequence (5'-3')
act-1 forward	TCCAAGAGAGGTATCCTTAC
act-1 reverse	CGGTTAGCCTTTGGATTGAG
daf-16 forward	CTTCAAGCCAATGCCACTACC
daf-16 reverse	GGAGATGAGTTGGATGTTGATAGC
skn-1 forward	GACGTCAATTTATGGAGTGTCG
skn-1 reverse	GAAGATGTTTTGTCGTGATCCG
sod-3 forward	CCAACCAGCGCTGAAATTCAATGG
sod-3 reverse	GGAACCGAAGTCGCGCTTAATAGT
gst-4 forward	TGGAGACTCATTGACTTGGG
gst-4 reverse	TCCTTTCTTGTTGCCACG
gst-10 forward	CGTGCCACAACTTTACTACTTC
gst-10 reverse	CAA CTG ACC AAG GAG CAT TC
